# Emerging predictors of femoral artery occlusion after pediatric cardiac catheterization

**DOI:** 10.1038/s41598-020-70891-5

**Published:** 2020-08-19

**Authors:** Lei Kou, Qian Wang, Whitney Annie Long, Feng Tang, Lei Li

**Affiliations:** 1 Department of Vascular Surgery, Beijing Anzhen Hospital, Capital Medical University, Beijing, 100029 China; 2 Department of Vascular Surgery, First Hospital of Tsinghua University (Beijing Huaxin Hospital), No.6 1st Jiuxianqiao Street, Chaoyang District, Beijing, 100016 China; 3 School of Clinical Medicine, Tsinghua University, Beijing, 100084 China

**Keywords:** Interventional cardiology, Peripheral vascular disease, Paediatric research, Risk factors

## Abstract

The Objective was to review the prevalence of femoral artery occlusion (FAO) after cardiac catheterization in children up to 12 years old from two centers in China and identify its related risk factors. After collecting clinical data from patients who had undergone pediatric cardiac catheterization, univariate and multivariate analysis were used to evaluate the correlations between FAO and clinical factors, including sex, age, height, weight, sheath size, operation time, therapeutic strategy, sheath/age, sheath/height and sheath/weight. The ROC curve was also used to assess the influence of risk factors to predict FAO. FAO occurred in 19 (0.9%) out of 2,084 children following cardiac catheterization. Patients with younger age, lower height, longer operation time, electrophysiological (EP) diagnosis or/and therapy for arrhythmias, higher Sheath/Age, higher Sheath/Height and higher Sheath/Weight ratios had higher risk for FAO compared to their respective control groups (*p* < 0.05). In the multivariate analysis, sheath/age and operation time were independent risk factors for FAO. Patients with operation time > 77.5 min or sheath/age > 0.5334 had a significantly higher risk for FAO. Operation time and sheath/age were confirmed as significant and independent risk factors associated with FAO. Operation time > 77.5 min and sheath/age > 0.5334 could effectively predict high risk of FAO after pediatric cardiac catheterization.

## Introduction

Percutaneous cardiac catheterization in children has been widely accepted since 1962^1–3^ , although it is an invasive procedure with several associated complications. Femoral artery occlusion (FAO) is one of the most common relevant complications with reported incidences of 0.7–11.4% in children^4–12^. A variety of relevant factors have been identified for FAO^7,10,12–14^, including age, weight, cyanosis, size of needle and sheath, puncture frequency, number of procedural catheter changes and total procedure time.

However, there remains uncertainty as to precise risk factors since publications represent simple case reports and different study designs and criteria. Furthermore, the literature lacks many large cohort studies which would allow calculation of the incidence of FAO and related risk factors after pediatric cardiac catheterization in China.

The objective of this study was to review the prevalence of FAO in children up to 12 years old after cardiac catheterization and identify risk factors associated with FAO in two centers.

## Materials and methods

### Materials

A database of 2,084 pediatric cardiac catheterization procedures performed via femoral artery cannulation at the First Hospital of Tsinghua University (Beijing Huaxin hospital) and Beijing Anzhen Hospital of Capital Medical University during the period August 1, 2009, through September 30, 2016 was reviewed in this study. Patients who had undergone the procedure were 12 years of age or younger. Cardiac catheterizations via venous approach alone had been excluded. Ultrasound guided puncture and closure device were not used in any cases. Data extraction was approved by our institution’s ethics committee (Biomedical research ethics committee, Beijing Huaxin hospital). The need to obtain informed consent from the patients was waived by the approving committee. The experiment including any relevant details was approved by the hospital ethics committee and performed in accordance with applicable guidelines and regulations. Human participants' names and other HIPAA identifiers were removed from all sections of the manuscript.

### Methods

All catheterization procedures were performed in accordance with routine procedure. Systemic heparinization (50–100 units/kg) was used during the procedure in all cases. Our protocols required hand pressure to the wound for at least 10 min after sheath extraction, until cutaneous bleeding ceased, followed by a pressurized bandage for 6 h. During this time, the patient was continuously observed for the presence of limb ischemic symptoms and if so, an ultrasound examination was performed. The presence of FAO was confirmed by ultrasound, in patients with at least one of the following symptoms: (1) decreased or absent palpable pedal pulses, (2) pale and cold lower extremities, (3) lower percutaneous oxygen saturation and blood pressure of the lower extremities.

Clinical factors including sex, age (years = days from birth/365), height (cm), weight (kg), sheath size (French), operation time (min), therapeutic strategy, ratio of sheath size to age (sheath/age), ratio of sheath size to height (sheath/height), ratio of sheath size to weight (sheath/weight) and FAO for children who undertook cardiac catheterizations were collected and analyzed in this study.

Therapeutic strategy of cardiac catheterization included electrophysiological (EP) diagnosis or/and therapy for arrhythmias, interventional therapy (IT) for congenital heart disease (CHD) and cardioangiography (CA) for CHD.

### Data analysis

Statistical analysis was performed with SPSS software (IBM, version 23.0). Data was presented as percentages and medians [Interquartile range (IQR)]. Mann Whiney test and chi-square analysis were used to evaluate the correlations between FAO and clinical factors. Independent risk factors for FAO were analyzed using multiple logistics regression analysis, with a p-value of < 0.10 used for the model selection of entry and a p-value of < 0.05 was necessary for retention. The ROC curve was used to assess the influence of risk factors to predict FAO and chi-square analysis was used to confirm the correlations between FAO and risk factors. A p-value < 0.05 was considered statistically significant.

## Results

### Patient characteristics

During the period of August 1, 2009 to September 30, 2016, a total of 2,084 pediatric cardiac catheterizations were performed at the First Hospital of Tsinghua University and Beijing Anzhen Hospital of Capital Medical University. The study population consisted of 826 males and 1,258 females, with a median age of 3.7 years old (IQR: 1.9–6.3 years old), a median height of 100 cm (IQR: 85–118 cm) and a median body weight of 15.3 kg (IQR: 11.2–21.0 kg). A wide range of sheath sizes from 4 to 10 French, with a median of 5 French were used. The median operation time was 60 min (IQR: 45–60 min). There were 290 EPs, 806 ITs and 988 CAs performed. Femoral artery occlusion occurred in 19 (0.9%) out of 2,084 children following cardiac catheterization. All patients with FAO were treated with heparin and 6 patients were treated with urokinase thrombolytic therapy. All patients experienced remission of symptoms with restoration of a normal pulse, which was confirmed by ultrasound. No FAO occured among the 37 patients who underwent repeat catheterizations in the same femoral artery. Patients with FAO had no heparin resistance during subsequent testing, whose immediate activated clotting time of whole blood (ACT) after operation is greater than 200 s. The median sheath/age was 0.31 (0.19–0.56). The median sheath/height was 5.26 (4.59–6.17). The median sheath/weight was 0.34 (0.25–0.45). Characteristics of these children are shown in Table 1.

**Table 1:** Demographic and patient characteristics undergoing cardiac catheterization during study period.

Characteristics	n = 2084^a^
**Sex**	
Male	826 (39.6%)
Female	1,258 (60.4%)
Age (years)	3.7 (1.9–6.3)
Height (cm)	100 (85–118)
Weight (kg)	15.3 (11.2–21.0)
Sheath size (French)	5 (5–5)
Operation time (min)	60 (45–60)
**Therapeutic strategy**	
EP	290 (13.9%)
IT	806 (38.7%)
CA	988 (47.4)
Sheath/age	0.31 (0.19–0.56)
Sheath/height	5.26 (4.59–6.17)
Sheath/weight	0.34 (0.25–0.45)
FAO	19 (0.9%)

*EP* electrophysiological diagnose or/and therapy for arrhythmias, *IT* interventional therapy for CHD, *CA* cardioangiography for CHD, *FAO* femoral artery occlusion.

^a^Data was presented as percentages and medians [Interquartile range (IQR)].

### The correlation between clinical factors and femoral artery occlusion after pediatric cardiac catheterization

These data are recorded in Table 2. Patients with younger age, lower height, longer operation time, EP, higher sheath/age, higher sheath/height and higher sheath/weight had a significantly higher risk for FAO compared to their respective control groups (*p* < 0.05). By multivariate analysis, sheath/age and operation time were independent risk factors for FAO (Table 3). Height was not included into the multivariate model because it was linearly dependent on age.

**Table 2:** The correlation between clinical factors and FAO after pediatric cardiac catheterization.

Characteristics	FAO (n = 19)^a^	No FAO (n = 2065)^a^	*p *value
**Sex**			0.825
Male	8 (42.1%)	818 (39.6%)	
Female	11 (57.9%)	1,247 (60.4%)	
Age (years)	2.3 (1.3–3.2)	3.7 (1.9–6.3)	0.009
Height (cm)	90 (80–103)	101 (85–118)	0.044
Weight (kg)	12.2 (10.0–16.1)	15.3 (11.2–21.0)	0.055
Sheath size (French)	5 (5–7)	5 (5–5)	0.237
Operation time (min)	80 (60–145)	60 (45–60)	0.001
**Therapeutic strategy**			0.009
EP	8 (42.1%)	282 (13.7%)	
IT	4 (21.1%)	802 (38.8%)	
CA	7 (36.8%)	981 (47.5)	
Sheath/age	0.63 (0.38–0.8)	0.31 (0.19–0.56)	0.001
Sheath/height	6.10 (5.00–7.37)	5.26 (4.59–6.17)	0.014
Sheath/weight	0.43 (0.31–0.50)	0.34 (0.25–0.45)	0.023

*EP* electrophysiological diagnose or/and therapy for arrhythmias, *IT* interventional therapy for CHD, *CA* cardioangiography for CHD, *FAO* femoral artery occlusion.

^a^Data was presented as percentages and medians [Interquartile range (IQR)].

**Table 3:** The correlation between clinical factors and FAO (Logistic regression model).

Variables	Wald	OR	95% CI	*p* value
Age (years)	2.133	0.778	0.555–1.090	0.144
Operation time (min)	22.026	1.021	1.012–1.029	0.000
Treatment	2.634	0.459	0.179–1.175	0.105
Sheath/age	8.392	5.435	1.729–17.087	0.004
Sheath/height	2.013	0.531	0.222–1.273	0.156
Sheath/weight	0.029	1.849	0.002–2,219.294	0.865

*FAO* femoral artery occlusion.

### The influence of risk factors to predict femoral artery occlusion after pediatric cardiac catheterization

**Figure 1:**
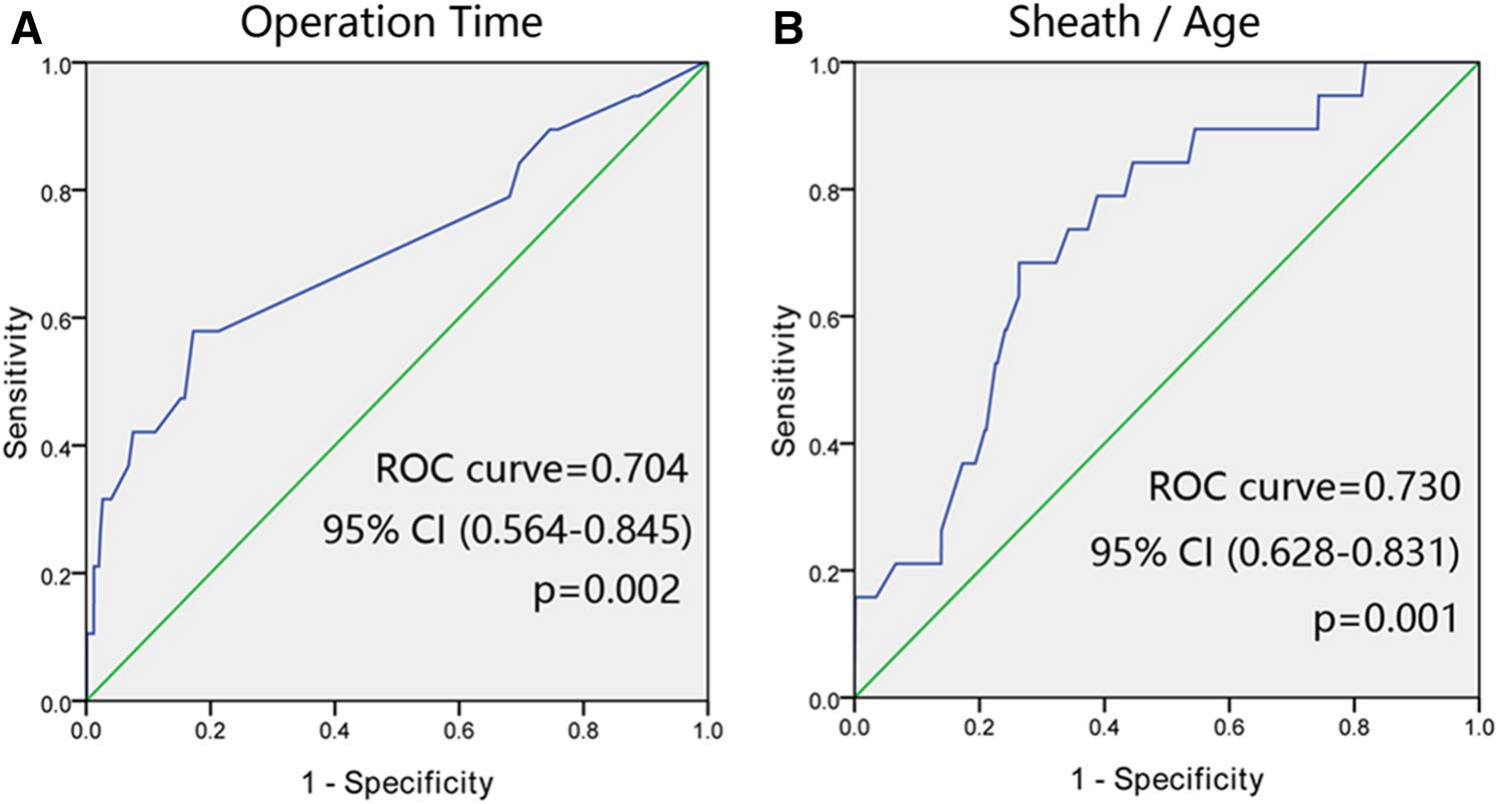
ROC curves for Operation time and Sheath/Age to predict the FAO after pediatric cardiac catheterization. (**A**) The area under the ROC curve of operation time was 0.704 (95% CI 0.564–0.845, p = 0.002); the optimal cutoff which simultaneously maximized both the sensitivity and specificity of the test was 77.5 min. (**B**) The area under the ROC curve of Sheath/Age was 0.730 (95% CI 0.628–0.831, p = 0.001); the optimal cutoff which simultaneously maximized both the sensitivity and specificity of the test was 0.5334.

To predict the occurrence of FAO, the influence of risk factors as mentioned above was assessed, and the ROC curve was plotted (Fig. 1). The area under the ROC curve of operation time was 0.704 (95% CI 0.564–0.845, *p* = 0.002) and the optimal cutoff which simultaneously maximized both the sensitivity and specificity of the test was 77.5 min. The area under the ROC curve of sheath/age was 0.73 (95% CI 0.628–0.831, *p* = 0.001) and the optimal cutoff which simultaneously maximized both the sensitivity and specificity of the test was 0.533. Racial/ethnic data were not available for this cohort.

The patients were then divided into two groups by the cutoff and chi-square analysis was used to confirm the relationship of operation time and sheath/age with FAO. The results showed that patients with operation time > 77.5 min or sheath/age > 0.5334 had a significantly higher risk for FAO (Table 4).

**Table 4:** The relationship of operation time and sheath/age with FAO.

Characteristics	FAO, N (%)	No FAO, N (%)	*p* value
**Operation time (min)**			0.000
≤ 77.5	8 (0.5%)	1709 (99.5%)	
> 77.5	11 (3.0%)	356 (97.0%)	
**Sheath/age**			0.000
≤ 0.5334	6 (0.4%)	1521 (99.6%)	
> 0.5334	13 (2.3%)	544 (97.7%)	

*FAO* femoral artery occlusion.

## Discussion

Femoral artery occlusion remains one of the most common complications despite the progress made in technique, equipment and peri-operative management of pediatric cardiac catheterization since 1974^1–8,11,15^.

FAO rarely occurs in the adult with normal femoral artery after cardiac catheterization^16^, but it has been reported to occur in 0.7–11.4% of children^4–10,12,15^. However the wide variation in patient age in the above articles, was associated with different incidences of FAO. Therefore, patients were limited to those under 12-years of age in our study. Girod et al.^4^ noted that the overall incidence of femoral artery thrombosis was 11 of 1,316 (0.8%) in 1982, Mehta et al.^8^ reported that there were 218 FAOs out of 11,073 (1.97%) children after cardiac catheterization during an 11-year study period. Despite a wide range of FAO incidence reported in the literature, we found that the prevalence of FAO was very low in large sample size studies. Our result showed that the incidence of FAO is 0.9%, which is consistent with the above literature reports.

Cardiac catheterization in children has unique pathophysiology^11^, with both spasm and injury proposed as mechanisms for FAO^17,18^. Franken et al.^19^ argued that the principal cause of femoral artery spasm was catheter size, showing that the incidence of femoral artery spasm increased significantly when the residual lumen of the femoral artery after catheterization was less than 55%. Glatz et al.^17^ thought that the ratio of the outer diameter of the arterial catheter to the size of the femoral artery was an important risk factor for arterial injury. They believed that body weight was a better surrogate for femoral artery size and reported that weight category and sheath size were independent risk factors of FAO by multiple logistic regression. Lopez Alvare and his colleagues^20^ estimated the diameter of the femoral artery for 125 pediatric patients by vascular ultrasound, and found that the diameter of the femoral artery was significantly associated with the age, height and body weight of the patients. Therefore, we calculated sheath/age, sheath/height and sheath/weight as variables to analyze the risk factors of FAO in our study. On univariate analysis, we found that higher sheath/age, higher sheath/height and higher sheath/weight all had higher risk for FAO compared to respective control groups (all *p* < 0.05), among which sheath/age had the greatest statistical significance. In the multivariate analysis, sheath/age was an independent risk factor for FAO. We also assessed the value of sheath/age to predict FAO by ROC curve. The area under the ROC curve of sheath/age was 0.73 (95% CI 0.628–0.831, *p* = 0.001) and the optimal cutoff was 0.5334. The above results were also proved by subsequent chi-square test. Therefore, we suggest that sheath/age could effectively predict high risk of FAO after pediatric cardiac catheterization in children (3.1), which has not been reported before.

Several other risk factors^7,8,10,12,14,15,17,18,21–24^ are reported to be associated with FAO after pediatric cardiac catheterization, including age, weight, cyanosis, size of needle and sheath, puncture frequency, number of procedural catheter changes, total procedure time andtherapeutic intervention. Vitiello and his colleagues^7^ suggested that age was an independent risk factor in infant femoral artery thrombosis. Glatz et al.^17^ reported that larger catheter size was an independent risk factor that significantly associated with FAO in a multivariate model. In a study by Lee et al.^23^, operation time was considered to be independently associated with FAO. One review^22^ identified independent variables which could increase the risk of FAO, including age < 3 years, type of therapeutic intervention and the use of a ≥ 6F guiding catheter. Some reports^10,14,15,24^ mentioned that children with low body weight were more prone to thrombosis. However, no other predictive risk factors listed above were found in our study except for operation time. This may result from the improvement of interventional equipment and puncture technology compared with older studies.

Vitiello and his colleagues found that the IT procedure was responsible for many of the FAO (43%) cases in their study. Rohit Mehta et al.^8^ reported that over half the arterial injuries were associated with an IT procedure (53%), thus EP only accounted for a small part. However, our study showed that EP was more prone to FAO, in which nearly half of the FAO occurred (42.1%). Long operation time and inadequate anticoagulation may lead to thrombosis. This may be the reason why EP accounted for more FAO after cardiac catheterization, since the operation time of EP was longer than the other two treatments in our study. Additionally, EP usually requires a larger sheath size than the other two treatments, and often requires multiple vascular approaches, such as unilateral or bilateral femoral vein puncture simultaneously. However, in the multivariate model, type of procedure was not found to be independently associated with FAO.

Ours is the first study which has assessed the influence of risk factors on FAO after pediatric cardiac catheterization by using ROC curve. Subsequently, the cutoff was calculated and the chi-square test was applied to verify the results, indicating that children who undertaken cardiac catheterization with operation time > 77.5 min (0.5% FAO in operation time ≤ 77.5 min vs. 3% in operation time > 77.5 min, p = 0.000) or sheath/age > 0.5334 (0.4% FAO in ratio ≤ 0.5334 vs. 2.3% in ratio > 0.5334, p = 0.000) had significantly higher risk for FAO. Therefore, the operation time should be shortened as much as possible, or heparin should be appropriately added and activated clotting time should be monitored when the operation time is prolonged to maintain the heparinization effect and reduce the risk of thrombosis. Moreover, the external diameter of the catheter for interventional or electrophysiological therapy should be minimized, which may challenge device developers. Furthermore, intraoperative and postoperative monitoring of ipsilateral limb oxygen saturation could be helpful for timely detection of FAO, as well as postoperative ultrasound^5^. Finally, prophylactic antispasmodic therapy in pediatric cardiac catheterization may be useful because spasm is one of the most important mechanisms of FAO^17,18^, especially for children with high risk of FAO.

The limitation of this retrospective study is the database of only two hospitals, which derived from their medical records. Despite our attempts to identify risk factors such as puncture frequency, number of procedural catheter changes, and specific type of therapy of cardiac catheterization, only partial data existed. In the future, further prospective studies with complete patient data will be needed to draw definitive conclusions or create a new predictive scoring system for FAO in children.

## Conclusion

In this study, operation time and sheath/age were confirmed as significant and independent risk factors associated with FAO after pediatric cardiac catheterization. Operation time > 77.5 min and sheath/age > 0.5334 could effectively predict high risk of FAO after pediatric cardiac catheterization.

### Consent for publication

Consent for publication was obtained for every individual person’s data included in the study.

## Data Availability

The datasets generated during and/or analysed during the current study are available from the corresponding author on reasonable request.
